# Effect of respiratory inhibitors and quinone analogues on the aerobic electron transport system of *Eikenella corrodens*

**DOI:** 10.1038/s41598-021-88388-0

**Published:** 2021-04-26

**Authors:** Rubén D. Jaramillo-Lanchero, Paola Suarez-Alvarez, Luis Teheran-Sierra

**Affiliations:** 1 Grupo de Investigación de Biomembranas (GIBIOM), CIFACS, Facultad Ciencias de La Salud, Centro Seccional de Investigación (CIUL), Universidad Libre Campus Barranquilla, Barranquilla, Colombia; 2 Grupo de Micología, Departamento de Microbiología, Facultad de Medicina, Universidad de Cartagena, Campus Zaragocilla, Cartagena, Colombia; 3 School of Agricultural and Veterinary Sciences, Technology Department, São Paulo State University (Unesp), Via de Acesso Prof. Paulo Donato Castellane s/n, Jaboticabal, SP 14884-900 Brazil

**Keywords:** Biochemistry, Microbiology

## Abstract

The effects of respiratory inhibitors, quinone analogues and artificial substrates on the membrane-bound electron transport system of the fastidious β-proteobacterium *Eikenella corrodens* grown under O_2_-limited conditions were studied. NADH respiration in isolated membrane particles were partially inhibited by rotenone, dicoumarol, quinacrine, flavone, and capsaicin. A similar response was obtained when succinate oxidation was performed in the presence of thenoyltrifluoroacetone and N,N’-dicyclohexylcarbodiimide. NADH respiration was resistant to site II inhibitors and cyanide, indicating that a percentage of the electrons transported can reach O_2_ without the *bc*_1_ complex. Succinate respiration was sensitive to myxothiazol, antimycin A and 2-heptyl-4-hydroxyquinoline-N-oxide (HQNO). Juglone, plumbagin and menadione had higher reactivity with NADH dehydrogenase. The membrane particles showed the highest oxidase activities with ascorbate-TCHQ (tetrachlorohydroquinone), TCHQ alone, and NADH-TMPD (N,N,N’,N’-tetramethyl-*p*-phenylenediamine), and minor activity levels with ascorbate-DCPIP (2,6-dichloro-phenolindophenol) and NADH-DCPIP. The substrates NADH-DCPIP, NADH-TMPD and TCHQ were electron donors to cyanide-sensitive *cbb'* cytochrome *c* oxidase. The presence of dissimilatory nitrate reductase in the aerobic respiratory system of *E*. *corrodens* ATCC 23834 was demonstrated by first time. Our results indicate that complexes I and II have resistance to their classic inhibitors, that the oxidation of NADH is stimulated by juglone, plumbagin and menadione, and that sensitivity to KCN is stimulated by the substrates TCHQ, NADH-DCPIP and NADH-TMPD.

## Introduction

The oral microbiological ecosystem of humans is extremely dynamic and consists of a complex system with various metabolic activities. Over 400 dissimilar bacterial species have been found on oral surfaces^1,2^ at a relatively constant temperature (34 to 36 °C) and a pH close to neutral in most areas, thus supporting the growth of a wide variety of microorganisms. *Eikenella corrodens* is commonly isolated from the human oral cavity and upper respiratory tract, and belongs to the family *Neisseriaceae*, genus *Eikenella*, and β-subdivision of the class Proteobacteria. This facultative anaerobic species is a gram-negative, fastidious, rod-shaped bacterium, and opportunistic pathogen in non-oral infections^3^.

In general, the flow of electrons in respiration is branched, comprising different dehydrogenases, quinones, *bc* complexes, haeme-copper respiratory oxidases, reductases and respiratory supercomplexes^4,5^. The expression of cytochromes and their complexes depends on environmental conditions such as the culture medium composition and oxygen gradient. In the electron transport systems of the prokaryotes and mitochondria, inhibitors of different respiratory complexes have been used, and terminal oxidases are characterized with artificial substrates, such as ascorbate-TMPD, ascorbate-DCPIP^6,7^, ascorbate-TCHQ^8^ and TCHQ, which exhibit different redox potentials. In addition, it is postulated that oxidized TCHQ can be spontaneously reduced again by NADH or NADPH^9,10^.

A scheme of the respiratory chain *E*. *corrodens* ATCC 23834, grown under O_2_-limited conditions^11^, consists of succinate, NADH and formate dehydrogenases, a ubiquinone, a cytochrome *bc*_1_ complex, and a KCN-sensitive *cbb*-type cytochrome *c* oxidase. Furthermore, previous studies^12,13^ showed that *E. corrodens* grows using nitrate as a possible alternative electron acceptor in the respiratory system. In this work, we studied the effect of inhibitors on the respiratory rate in the presence of endogenous substrates of the electron transport chain in isolated membranes from *E. corrodens*, cultured under O_2_-limited conditions. Additionally, the effects of naphthoquinone and ubiquinone analogues on the respiration and nitrate reductase activity in membranes were analysed. Finally, *cbb*3 cytochrome c oxidase activity was determined with the use of different intermediaries. Here, the purpose is to elucidate the nature and functional organization of the respiratory chain components with respiratory inhibitors, quinone analogues and artificial substrates for terminal cytochrome *c* oxidase.

## Results

The effect of electron transport inhibitors in isolated membranes from *E*. *corrodens* was determined**.** The NADH dehydrogenase (NDH) and succinate dehydrogenase (SDH) activities were similar (Table 1); however, NADH-coupled respiratory oxidase activity was 2.4-fold lower than that of succinate oxidation (Table 1). This differential finding suggests by a low expression of NDH at the tested growth conditions. In bacteria, the presence of one of the three groups of respiratory NDHs has been reported^17,18^. The inhibitors of NDH-1 and NDH-2 such as rotenone, quinacrine, dicoumarol and flavone (inhibitor of NDH-2) inhibited NADH oxidation by 30–40% (Table 2). HOQNO, antimycin A, myxothiazol and cyanide were poor inhibitors of NADH dependent O_2_, with an inhibition of 31% with HQNO and 16–18% with the other inhibitors (Table 2). A similar response also was observed for an antimycin A plus myxothiazol experiment under, “double kill” conditions^19^. These results suggest a very low oxidation of NADH, which does not allow differentiation of the type of NDH and show marginal use of the *bc*_1_ complex.

**Table 1:** Respiratory activities associated with cell membranes of *Eikenlla corrodens* ATTC 23834 grown below aerobic static conditions.

Substrates	Oxidase^a^	Oxidoreductase^b^
Succinate	50 ± 5	200 ± 15
NADH	21 ± 2	235 ± 13
NADH-DCPIP	110 ± 7	–
NADH-TMPD	130 ± 10	–
Ascorbate	12 ± 1	–
Ascorbate-DCPIP	80 ± 5	–
Ascorbate-TMPD	135 ± 12	–
TCHQ	195 ± 15	–
Ascorbate-TCHQ	340 ± 20	–
Nitrate reductase	–	230 ± 10^c^

^a^The oxidases were measured polarographically with a Clark-type electrode as described in Material and methods. Oxidase activities are expressed in nanomoles of O_2_ min^−1^ mg protein^−1^.

^b^Dehydrogenase activities are presented in nanomoles of reduced DCPIP min^−1^ mg protein^−1^.

^c^Nitrate reductase activity expressed as nanomoles of oxidized methyl viologen min^−1^ mg protein^−1^.

Data were processed with the aid of GRAFIT version 4 (Erithacus software) and OriginPro Sr2.

**Table 2:** Effect of electron transport inhibitors on NADH- and succinate-dependent respiration^a^ in cell membranes of *Eikenella corrodens* ATCC 23834.

Inhibitor	Concentration (μM)	Percent Inhibition	
		NADH	Succinate
Rotenone	300	41 ± 3	–
Dicoumarol	250	38 ± 2	–
Quinacrine (Atebrine)	300	36 ± 2	–
Flavone	300	32 ± 3	–
TTFA	100	–	13 ± 3
DCCD	50	–	22 ± 2
HQNO	300	31 ± 2	59 ± 3
Antimycin A	300	16 ± 2	64 ± 2
Myxothiazol	30	18 ± 3	89 ± 3
KCN	50 or 100	15.5 ± 2.5	90 ± 2
Antimycin A plus Myxothiazol	300/30	20 ± 2.7	90 ± 2

^a^The oxidases were measured polarographically with a Clark-type electrode as described in Material and methods. The specific activities registered in absence of inhibitors were 21 ± 2 and 50 ± 5 nmol O_2_ min^−1^ mg protein^−1^ for NADH and succinate oxidase, respectively. Data were processed with the aid of GRAFIT version 4 (Erithacus software) and OriginPro Sr2.

**Figure 1:**
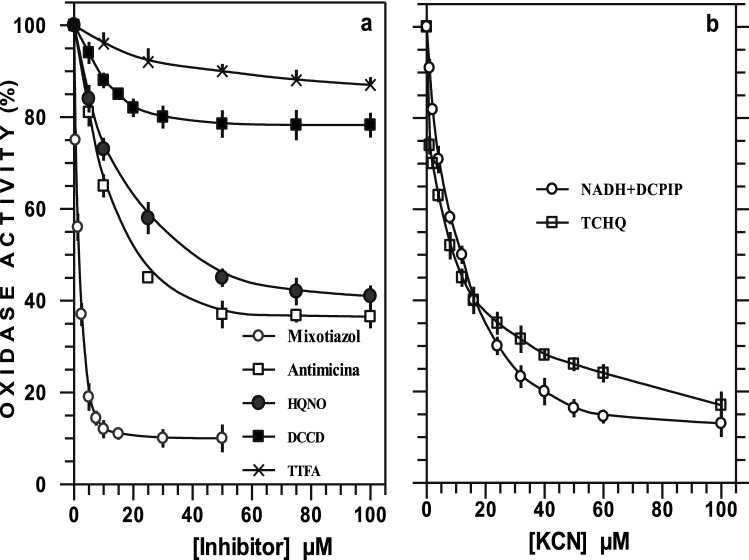
Effects of respiratory inhibitors on oxidase activities in membranes of *E. corrodens*. Cells were grown for 20 h under oxygen-limited conditions. Activities were measured and inhibitors added as described in Materials and methods. (**A**) Effect of myxothiazol, antimycin A, HQNO, DCCD and TTFA on succinate oxidase activity. (**B**) Effect of KCN on the NADH-DCPIP and TCHQ oxidases. The specific activities registered in absence of inhibitor were 95 ± 5, 110 ± 9 and 181 ± 15 nmol O_2_ ∙ min^−1^ ∙ mg protein^−1^ for succinate, NADH plus DCPIP and TCHQ oxidases, respectively. Data were processed with the aid of GRAFIT version 4 (Erithacus software) and OriginPro Sr2.

**Figure 2:**
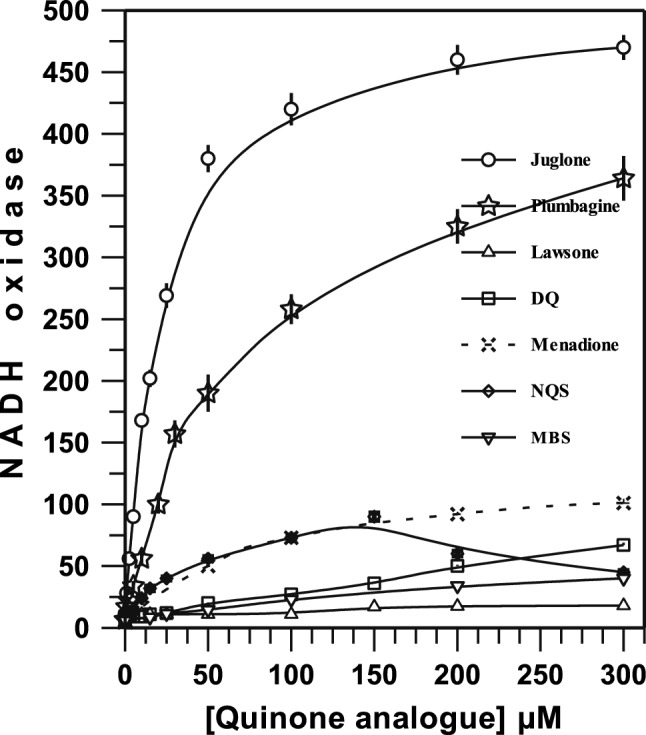
Stimulation of NADH oxidase in membranes from *E. corrodens* ATCC 23834 with juglone, plumbagine, menadione, NQS, MBS, lawsone and DQ. The oxidation rates were measured as described in Materials and methods. The specific activities registered in absence of quinone analogues was 4.5 ± 1.5 nmol O_2_ ∙ min^−1^ ∙ mg protein^−1^. Data were processed with the aid of GRAFIT version 4 (Erithacus software) and OriginPro Sr2.

The effect of *bc*_1_ complex inhibitors on succinate-dependent respiration is shown in Fig. 1A. Succinate-dependent respiration was inhibited by 60% by antimycin A and HQNO at the same concentration (Fig. 1A and Table 2). Succinate oxidation was found to be more sensitive to inhibition by myxothiazol than by HQNO and antimycin A. Succinate respiration was inhibited with a half-maximal inhibitory concentration (IC_50_) value in the presence of 1.7 µM myxothiazol, and O_2_ consumption was abolished at 30 µM myxothiazol. The IC_50_ concentrations were of 20 µM antimycin and 40 µM HQNO. The results indicated that the *bc*_1_ complex (complex III) of *E*. *corrodens*^11^ was more sensitive to low concentrations of myxothiazol than to antimycin and HQNO. Succinate oxidation was only inhibited 10–15% by TTFA and DCCD at 100 µM (Fig. 1A), and 2,4-dinitrophenol caused no inhibition (data not shown). The data suggest a partial inhibition of NDH in the presence of rotenone, quinacrine, dicoumarol, flavone and *bc*_1_ complex inhibitors. Finally, the results suggest that SDH was weakly inhibited by TTFA, and the results were similar for *Bacillus subtilis* and *B*. *cereus*^15,20^.

Regarding the behaviour of the terminal oxidase in membranes of *E*. *corrodens*, the specific oxidase activities for various substrates in isolated membrane particles were determined (Table 1). The rate of O_2_ uptake with NADH plus TMPD (E ~  =  + 260 mV at pH 7.0) was 2.4- and 1.6-fold faster than that with NADH plus DCPIP (E ~  =  + 217 mV at pH 7.0), and ascorbate plus DCPIP (E = 58 mV at pH 7.0), respectively, but the activity levels of the NADH- and ascorbate-TMPD oxidases (Table 1) were similar. The oxidation of TCHQ alone (E = 350 mV pH 7.0) was 1.6-fold faster than that with NADH plus TMPD and ascorbate-TMPD. In the presence of ascorbate, TCHQ was oxidized at higher rates than the previous substrates. Moreover, the specific activities of oxidoreductase were determined. The SDH and NDH activities (Table 1) were 200 and 235 nmol reduced DCPIP · mg protein^−1^ · min^−1^, respectively, but the SDH-DCPIP was 35 nmol reduced DCPIP · mg protein^−1^ · min^−1^. In the SDH assay was utilized to the phenazine methosulfate (PMS) was utilized as the mediator and DCPIP was utilized as final acceptor, while in the second assay, endogenous ubiquinone was utilized as the mediator. Thus, compared to other mediators, PMS is an order of magnitude more efficient as a direct electron acceptor for SDH. Furthermore, the specific activity of nitrate reductase in isolated membranes of *E*. *corrodens* was determined in the presence of methyl viologen (MV) reduced by dithionite as an electron donator, and the activity was 230 nmol oxidized MV · mg protein^−1^ · min^−1^. The above experiments indicated that the rate of oxidation with NADH in the presence of DCPIP and TMPD was higher than that with NADH alone (Table 1), which is in agreement with the role of DCPIP and TMPD as electron donors at the respiratory chain downstream from ubiquinone^6^. Moreover, TCHQ alone and TCHQ with ascorbate were oxidized, but showed specific activities higher than those of physiological substrates and artificial substrates (Table 1). In *E*. *corrodens*, nitrate reduction has been demonstrated as a possible electron acceptor pathway to oxygen in the respiratory system^12,13^, and under our experimental conditions, we found the presence of membrane-bound activity of nitrate reductase by oxidation of MV.

Respiration with NADH oxidation in the presence of DCPIP or TMPD established a bypass of NDH at c-type cytochrome and/or cytochrome oxidase. Cyanide inhibition of NADH-DCPIP and TCHQ oxidase revealed a monophasic curve in both cases (Fig. 1B). Respiration with NADH-DCPIP and TCHQ oxidation with IC_50_ of 12 and 9 µM KCN, respectively, and 100 µM KCN caused more than 80% inhibition. Likewise, the NADH-TMPD dependent activity was 80% inhibited by 100 µM KCN. In accordance with these results, we previously reported that succinate and ascorbate-TMPD oxidation involves a KCN-sensitive type-*cbb*´ cytochrome *c* oxidase^11^, with monophasic kinetics and IC_50_ values of 3 and 6 µM for cyanide, respectively. TCHQ was utilized to measure the quinol oxidase activities in *A*. *diazotrophicus* PAL5^21^ membranes and cytochrome *bo*_3_, highly purified from *Bacillus cereus* PYM1^22^; however, TCHQ, a benzoquinone similar to the endogenous ubiquinone of *E*. *corrodens*^11^, works in a more coherent way (physiological) and its oxidation proceeds via the *bc*_1_ complex pathway and/or KCN-sensitive cytochrome *c* oxidase. Consequently, these results indicated the presence of a KCN-sensitive terminal oxidase and one fraction with very low respiration (13–17%) remaining at high concentrations of cyanide (100 µM).

The effect of quinone analogues on the electron transport of membranes was determined. The respiratory activities in isolated membrane particles were examined by the addition of various naphthoquinone and ubiquinone analogues that differ in their structural features and redox potentials. The NDH and SDH activities were measured in the presence of water-soluble analogues of quinone (Table 3). Compared to the endogenous ubiquinone activity, the NDH: -juglone-, -plumbagine- and -menadione-DCPIP oxidoreductase activities were were increased 3-, 2- and 1.5-fold, respectively, with very high oxidoreductase activities (≥ 70%) remaining in the presence of site I inhibitors (data not shown). Succinate:juglone-DCPIP doubles the SDH-DCPIP oxidoreductase activity. These results showed that compared to the other analogues, juglone has a greater ability to catalyse the electron transfer from NADH or succinate to DCPIP. The different capacities of the three naphthoquinones as electron acceptors of dehydrogenases may be due to the chemical structure and redox potential of these quinone analogues.

**Table 3:** Activities of NADH and succinate: quinone-DCPIP oxidoreductases by different quinone analogues in membranes preparations of *Eikenella corrodens* ATCC 23834.

Quinone analogue added	Oxidoreductase activity^a^			
	NADH	%	Succinate	%
None	233.5 ± 6.5	100	36 ± 5^b^	100
Juglone	726 ± 6	311	69 ± 8	191.7
Plumbagine	488.5 ± 8.5	209	32 ± 3	89
Menadione	369 ± 3	158	33 ± 2	91.7
Lawsone	218.5 ± 3.5	93.6	36 ± 3	100
Duroquinone	251 ± 3	107.5	42 ± 4	116.7

^a^All quinone analogues were added at a final concentration of 300 μM. The specific activity for oxidoreductases is presented as nanomoles of reduced DCPIP min^−1^ mg protein^−1^.

^b^Succinate: endogenous ubiquinone-DCPIP oxidoreductase activity was measured in absence of PMS. Succinate dehydrogenase activity with PMS-DCPIP was 187 ± 7 nmol of reduced DCPIPmin^−1^ mg protein^−1^. Culture procedures and activity assays were performed as described in Materials and Methods.

The rate of NADH oxidation increased with the concentration of different quinone analogues (Fig. 2). The capacity of different quinone analogues to stimulate NADH oxidation varies. The highest NADH oxidation rates were obtained with juglone, plumbagine, menadione, sodium 1,2-naphtho-quinone-4-sulfonate (NQS), and duroquinone (2,3,5,6-tetramethyl-1,4-benzoquinone). Higher concentrations of 150 µM NQS caused partial diminution of stimulated respiratory activity, probably by the effect of sulfonate. Lower NADH oxidation activities were obtained with menadione bisulfite (MBS), lawsone and 1 mM decylubiquinone (data not shown). Apparent Km values were 21, 49 and 74 µM for juglone, plumbagine and menadione, respectively. The kinetics of stimulation by naphthoquinone analogues are in accordance with the data for *B*. *cereus*^14^ and *B*. *subtilis aroD*^23^. NADH-dependent respiration with quinone analogues was inhibited by rotenone, quinacrine, dicoumarol, capsaicin, flavone, site II inhibitors, SHAM (salicylic hydroxamate) and KCN (curves of inhibition at concentrations of 1–300 µM, data not shown) a similar form as in its absence (Table 2). NADH oxidation in the presence of menadione and juglone was 20–50% inhibited by AgCl (data not shown), a potent inhibitor of Na^+^-NQR^17^. Finally, succinate oxidation was not stimulated by quinone analogues, at concentrations of 1–400 µM (data not shown).

Our results indicated that juglone, plumbagine and menadione naphthoquinones are far more effective than endogenous ubiquinone in NADH oxidation. Additionally, the polarographic experiments suggest that the activation exerted by naphthoquinone analogues on NADH oxidase was coupled to an augmentation of electron transport through the cytochrome system. These results are in accordance with previous data in *Escherichia coli*, in which it was suggested that NDH I, ubiquinone and cytochrome oxidase do not produce significant amounts of superoxide anions and H_2_O_2_^24^, and ubiquinone is involved in defence against oxidative stress at the cytoplasmic membrane^25^. Finally, the results in the presence of site I inhibitors and quinone analogues in membranes of *E*. *corrodens* could not distinguish which types of respiratory NDH^17,18^ predominate under our experimental growth conditions.

## Discussion

To our knowledge, we reported the first study on the respiratory chain of E. corrodens^11^, and our results are in satisfactory agreement with the electron transport system of the genus *Neisseria*^26–29^ and genetic data on the *Eikenella corrodens* 23834 genome (https://www.ncbi.nlm.nih.gov/genome/2067?genome_assembly_id=172078). Data on the respiratory inhibitors, the effect of quinone analogues, and the nitrate reductase activity in membranes of *E*. *corrodens* ATTC 23834 are scarce. The *E*. *corrodens* genome shows the presence of NDH, SDH, *bc*_1_, oxidase, and reductase complexes. The SDH and NDH activities were similar; however, the activity of NADH oxidation under all titration was 2.4-fold lower than that of respiration with succinate. Our results demonstrate that NADH respiration is partially inhibited by rotenone, dicoumarol, quinacrine, flavone, HQNO (Table 2) and Ag + , suggesting that it may possess NDH-1 and/or Na^+^-NQR, as indicated by the genomic sequence of *Neisseria* sp^28,30^. Likewise, an nqr operon has been found in many marine and pathogenic bacteria^15,19^. In contrast, a significant inhibition of *N*. *gonorrhoeae* NADH oxidase^31^ was obtained with low concentrations of rotenone and HQNO (< 10 µM), which may reflect that NDH-1 and Na^+^-NQR are very sensitive to the inhibitors rotenone and HQNO^17,18^, respectively; however, in membranes of *N*. *meningitidis*^28^, NADH oxidation was weakly inhibited by rotenone, and highly sensitive to HQNO. Within the same family, the differences in sensitivity to the inhibitors of site I should be explained by the force of interaction with their active site for each case and/or growth conditions.

Our results suggest the resistance of SDH to TTFA inhibitors. Additionally, the preliminary analysis of the genome and amino acid sequence of complex II of the family *Neisseriaceae* (*N*. *shayeganii* 871, *N*. *weaver*, *N*. *arctica*, N. *meningitidis* serogroup B (strain MC58), *N*. *gonorrhoeae* (strain ATCC 700825/FA 1090)) and especially in *E*. *corrodens* indicate two hydrophobic subunits, C and D, suggesting succinate-ubiquinone reductase type C with a *b*-type haem (https://www.uniprot.org/uniprot/C0DU23; https://www.uniprot.org/uniprot/C0DU24). The succinate respiration was very sensitive to myxothiazol, antimycin A and HQNO (Fig. 1), and the *bc*_1_ complex of *E*. *corrodens* was more sensitive to low concentrations of myxothiazol than to antimycin and HQNO; in contrast, NADH oxidation was weakly inhibited by site II inhibitors and “double kill” conditions^19^. Our data obtained from inhibition by antimycin and HQNO, on physiological substrates in *E*. *corrodens*, taken together with the studies of *N*. *meningitidis* by Yu and DeVoe^28^ and *N*. *gonorrhoeae* by Kenimer and Lapp^32^, indicate that these inhibitors are similar, with the exception of succinate-dependent respiration in *N*. *gonorrhoeae*, where succinate oxidase cannot be inhibited by HQNO^31^.

We previously reported a high sensitivity of type-*cbb*' cytochrome *c* oxidase to cyanide in cytoplasmic membranes of *E*. *corrodens*^11^ in the presence of succinate- and ascorbate–TMPD-dependent substrates, and functional analysis of the genome showed the presence of a *cbb*_3_-type terminal oxidase (https://www.ncbi.nlm.nih.gov/ipg/?term=eikenella+corrodens+%5Borgn%5D+cytochrome-c+oxidase%2C+cbb3-type). NADH oxidase is far less affected by cyanide than succinate-dependent respiration. Our data described here indicate that cyanide inhibits NADH-DCPIP and TCHQ oxidases, exhibiting monophasic kinetics with IC_50_ values of 12 and 9 µM, respectively, and inhibits NADH-TMPD oxidase. The activity measured with TCHQ does not represent the maximum activity of the respiratory system of *E*. *corrodens*. The rate of oxidation of TCHQ possibly indicates a very efficient interaction with the *bc*_1_ complex. The TCHQ oxidation activity in membranes of *E*. *corrodens* is similar or greater than that reported for *A*. *diazotrophicus*^21^, *B*. *cereus*^22^ and *H*. *pylori*^33^. It has been accepted that the point of entry of electrons from TPMD and DCPIP into the respiratory system is at the *c*-type cytochrome level. In conclusion, KCN strongly inhibited respiration with succinate or with artificial substrates that preferentially feed the terminal part of the respiratory chain (ascorbate-TMPD and ascorbate-TCHQ); moreover, with somewhat less efficiency, mixtures of substrates open a bypass from the NDH dehydrogenase (NADH-DCPIP and NADH-TMPD). This result strongly suggests that under the growth conditions that we studied here, CN-sensitive oxidase is dominant in the respiratory system of *E*. *corrodens*. In addition, a possible interpretation with a background activity in the NADH-DCPIP and TCHQ oxidation of 13% and 17% with 100 µM cyanide, respectively can use a *bb*′-type oxidase with very low expression level in our growth conditions.

Likewise, the complete genome sequences of *N. meningitidis*^34^ and *N*. *gonorrhoeae*^25^ indicate that they contain a *cbb*' or *cbb*_3_ complex with IC_50_ values below 10 µM KCN in the presence of succinate as a respiratory substrate^28,32^; however, NADH oxidation in the membranes of *N*. *gonorrhoeae* appears to have an IC_50_ value of 22 µM KCN^32^. Nevertheless, very recently Osyezka et al^35^ reported that the interaction of cyanide with the native ferricytochrome *c*_1_ of photosynthesis bacterium *Rhodobacter capsulatus* cytochrome *bc*_1_ complex is an interesting new finding and suggests caution for viewing cyanide as a simple inhibitor of cytochrome oxidase.

It is clear that endogenous UQ is not an optimal mediator of electron transport between dehydrogenases and oxidases, which is especially critical in the oxidation of NADH and formate^11^. In this article, it is demonstrated that the addition of quinone analogues, especially juglone, plumbagine and menadione, to membranes from *E*. *corrodens* results in stimulation of NADH-dependent respiration. At the maximum levels of juglone, plumbagine and menadione, NADH oxidase activity was stimulated 54-, 43- and 12-fold, respectively. Even though the structures and redox potentials of naphthoquinones are very different from those of endogenous ubiquinone, they have higher reactivity with NDH. Furthermore, oxygen consumption apparently does not occur as a product of hydrogen peroxide formation, suggesting that electron transport occurs across the respiratory system. Very recently, Seaver and Imlay^25^ reported that H_2_O_2_ is primarily formed by a source outside the respiratory system. Thus, it would seem that the above quinone analogues are better electron acceptors for NDH than endogenous ubiquinone. The activity measured with TCHQ does not represent the maximum potential activity of electron transport in the respiratory system of *E*. *corrodens*. The rate of oxidation of TCHQ possibly indicates a very efficient interaction with *bc*_1_ complex. The TCHQ oxidase activity in membranes of *E*. *corrodens* is similar to or greater than that reported for *A*. *diazotrophicus*^21^, *B*. *cereus*^22^ and *H*. *pylori*^33^.

Previous studies showed that the metabolism of glutamate, serine and proline was associated with relatively high rates of nitrate reduction and the respiratory system in *E*. *corrodens*^12^. The amount of nitrate utilized was calculated on the basis of the nitrite level detected in culture filtrates from cells of *E*. *corrodens* grown aerobically. These findings suggest that the denitrification machinery is apparently not expressed, where nitrite is reduced to nitric oxide (NO), nitrous oxide, and, finally, dinitrogen; additionally, it seems that this organism does not express the pathway converting nitrite into ammonia by respiratory cytochrome c nitrite reductase sirohaeme containing NrfA or detoxifying enzyme NirBD^36^. Nitrite in bacteria is produced by one of three different types of nitrate reductases: periplasmic dissimilatory (Nap), membrane-associated respiratory (Nar) and soluble assimilatory (Nas). Gully and Rogers^12^ did not directly show whether the nitrate reductase is type Nap or Nar. This article is the first to demostrate the presence of membrane-bound respiratory activity of nitrate reductase (dissimilatory nitrate reductase, Nar) in *E*. *corrodens*, and the genome sequence shows the presence of nitrate reductase (https://www.ncbi.nlm.nih.gov/ipg/?term=eikenella+corrodens+%5Borgn%5D+nitrate+reductase). However, according to genomic information and studies other *Neisseria* species^26,30,34^ can express partial denitrification pathways, possessing genes necessary for the reduction of nitrite to nitrous oxide, via nitrite reductase AniA, and NO reductase NorB^37^, under limited oxygen conditions; and finally do not possess a known nitrate reductase^37^.

In summary, our data strongly indicate that NADH- and succinate-dependent respiration in membranes of *E*. *corrodens* ATCC 23834 is resistant to inhibitors of NDH and SDH. However, succinate respiration is very sensitive to inhibitors of complex III. Likewise, succinate, NADH-DCPIP, NADH-TMPD and TCHQ oxidase are electron donors for a cyanide-sensitive *cbb*' cytochrome c oxidase. However, NADH oxidase is resistant to site II inhibitors and cyanide, indicating that a percentage of the electron transported can possibly reach O_2_ without passing through the *bc*_1_ complex and a type *bb*′ oxidase with very low expression level. Juglone, plumbagine and menadione naphthoquinones, with different structures from that of endogenous ubiquinone, higher reactivity with NADH dehydrogenase. Finally, the presence of dissimilatory nitrate reductase in the respiratory system of *E*. *corrodens* ATCC 23834, grown under O_2_-limited conditions is demonstrated for the first time, which confirms the suggestions in previous studies^11,12^ about growth using nitrate as an alternative electron acceptor. *cbb*´ cytochrome *c* oxidase and nitrate reductase as terminal electron acceptors may be important determinants of pathogenicity in response to microaerobic conditions to permit the colonization of oxygen-limited environments and nitrate^27^. The nitrite formed may be an important substrate source for bacteria implicated in periodontal disease and other oral infections.

Future work should be done to clarify the electron transport chain of NDH towards the *bc*_1_ complex, and studies on the effect of oxidized and reduced benzoquinone analogues in the respiratory chain are necessary. Additionally, isolation and characterization of cytochrome complexes, and anaerobic respiratory systems areas undergoing investigation in our laboratory. The availability of the genomic DNA sequence for Neisseria sp. would facilitate the design of genomic probes to clone similar genes from *E*. *corrodens*; furthermore, the *Eikenella corrodens* ATCC 23834 whole genome shotgun (WGS) project is currently underway (https://www.ncbi.nlm.nih.gov/genome/2067?genome_assembly_id=172078); http://www.ncbi.nlm.nih.gov/sites/entrez?db=genomeprj&cmd=Retrieve&list_uids=30493).

## Materials and methods

### Cultures, cell disruption, and membrane preparation

*Eikenella corrodens* ATCC 23834 was grown under O_2_-limited conditions as described previously^11^. The final pH of the culture media was adjusted to 7.4 with NaOH. All cultures were maintained at 34 °C without shaking. Cells in the stationary phase of growth (20–24 h of growth) were harvested and washed twice with cold 50 mM Tris, 5 mM EDTA, and 0.2 M NaCl, pH 7.5 (TEN buffer). Procedures for cell disruption using ultrasonication, membrane isolation, and protein concentration determination were similar to those described by Jaramillo et al.^11^.

### Respiratory activities

Oxidase activities were determined polarographically at 34 °C as previously described^11,14^ using a Clark-type electrode covered by an ultra-thin Teflon membrane (YSI model 53 Oxygen-meter, Yellow Spring Instruments). Cytochrome oxidase activities were determined with 10 mM sodium ascorbate plus 0.1 mM TMPD at pH 6.8. In addition, oxygen consumption was determined in the presence of 10 mM ascorbate plus 0.08 mM DCPIP; with 0.5 mM NADH plus 0.1 mM TMPD and 0.5 mM NADH plus 0.08 mM DCPIP at pH 7.4; and with 10 mM ascorbate plus 3.5 mM TCHQ, and 3.5 mM TCHQ at pH 6.6. The experiments are means of at least 3 experiments.

### Respiratory inhibitor assay

The effect of inhibitors on the respiratory rate was evaluated polarographically and the compounds were dissolved as previously described^11^. Potassium cyanide and quinacrine were dissolved in 50 mM potassium phosphate pH 7.0; dicoumarol was dissolved in 30 mM KOH, and 2-heptyl-4-hydroxyquinoline-N-oxide (HQNO), antimycin A_3_, myxothiazol, thenoyltrifluoroacetone (TTFA), N,N’-dicyclohexylcarbodiimide (DCCD), 2,4-dinitrophenol, rotenone flavone, and capsaicin were dissolved in dimethyl sulfoxide (DMSO). The concentration of DMSO used did not affect the respiratory activities tested. These inhibitors were preincubated with membranes before the addition of the substrates.

### Quinone assays

The effect of quinone analogues on the respiration rate was measured in the polarographic experiments. Oxidase activities were determined in absolute ethanol solutions (0.025 ml or less) containing quinone derivatives that were added to the membranes 2–4 min before the beginning of NADH and succinate oxidase assays (controls were made with ethanol alone). In 50 mM phosphate buffer at pH 6.6 spontaneous oxidation of quinol analogues is minimal^14^. The quinone analogues were menadione, menadione sodium bisulfite (MBS), lawsone, plumbagine, juglone, sodium 1,2-naphtho-quinone-4-sulfonate (NQS), duroquinone (DQ), and decylubiquinone. Data are means of at least 3 experiments.

### Oxidoreductase activities

The SDH and NDH activities were determined essentially as described elsewhere^15^ in a DU640 Beckman spectrophotometer (Beckman Instruments, Fullerton, CA). The succinate: DCPIP oxidoreductase activity was measured at 30 °C in 1 ml of a mixture containing 100 mM potassium phosphate pH 7.4, membranes (0.1 mg of protein), 100 μM KCN, 40 mM disodium succinate, 1 mM PMS, and 0.08 mM DCPIP. The activity of NDH was measured under the same conditions, except that succinate and PMS were replaced by 0.2 mM NADH. An extinction coefficient of 21 mM^−1^ cm^−1^ was used for DCPIP. The nitrate reductase activity was measured based on the oxidation of reduced MV as described by Kučera^16^. The nitrate reductase activity was measured under anaerobic conditions in an assay mixture (2.5 ml) containing a N_2_-saturated solution of 0.1 mM sodium phosphate pH 7.4, 1 mM MV, and membranes (0.1 mg of protein). MV was reduced by addition of sodium dithionite. The reaction was started by the injection of an anaerobic solution of potassium nitrate (2 mM, final concentration). Oxidation of MV was monitored at 600 nm using an extinction coefficient of 11.4 mM^−1^ cm^−1^. Data are means of at least 3 experiments.
